# Metachronous head and neck osteosarcoma in an elderly patient: A rare case report with prior verrucous carcinoma

**DOI:** 10.1097/MD.0000000000045968

**Published:** 2025-11-21

**Authors:** Abolfazl Koozari, Mohammadreza Elhaie, Mohammad Reza Khademi

**Affiliations:** a Department of Medical Physics, School of Medicine, Ahvaz Jundishapur University of Medical Sciences, Iran; b Department of Medical Physics, School of Medicine, Isfahan University of Medical Sciences, Iran; c Department of Radiooncology, School of Medicine, Cancer Prevention Research Center, Seyyed Al-Shohada Hospital, Isfahan University of Medical Sciences, Iran.

**Keywords:** carcinoma, head and neck neoplasms, neoplasms, osteosarcoma, second primary, verrucous

## Abstract

**Rationale:**

Head and neck osteosarcoma is a rare malignancy, accounting for 6%–10% of all osteosarcomas, and typically affects younger individuals. Its occurrence as a metachronous tumor in elderly patients, particularly after radiotherapy, is exceedingly uncommon.

**Patient concerns:**

A 91-year-old man with a history of right lateral tongue verrucous carcinoma treated by surgery and adjuvant radiotherapy (50 Gy) in 2006 presented in 2024 with progressive hoarseness and dysphagia for 6 months.

**Diagnoses:**

Clinical examination revealed a firm neck mass. Laryngeal biopsy showed benign keratosis, while core needle biopsy demonstrated a malignant mesenchymal tumor with osteoid production. Immunohistochemistry showed negative cytokeratin and p63, and Ki-67 positivity in 20% of cells, confirming osteosarcoma. Imaging revealed a calcified lesion involving the hyoid, thyroid cartilage, and tongue base, consistent with metachronous head and neck osteosarcoma.

**Interventions:**

Due to advanced age and comorbidities (hypertension and diabetes), palliative radiotherapy (30 Gy in 15 fractions) was administered. Surgery and chemotherapy were avoided because of frailty and limited benefit.

**Outcomes:**

The patient tolerated treatment well, achieving partial symptom relief without major toxicity. Long-term follow-up continues with a focus on quality of life.

**Lessons:**

This case highlights the diagnostic challenges of secondary head and neck osteosarcoma following radiotherapy and the importance of long-term surveillance in elderly patients. Individualized management balancing treatment efficacy and tolerability is essential in geriatric oncology.

## 1. Introduction

Osteosarcoma is a primary bone malignancy predominantly affecting adolescents and young adults, with over 90% of cases occurring in the long bones of the appendicular skeleton.^[1–4]^ In contrast, head and neck osteosarcoma is rare,^[5]^ accounting for 6% to 10% of all cases,^[6]^ and presents distinct clinical and histopathological features.^[5,7,8]^ Its incidence in elderly patients is exceptionally low, and its emergence as a metachronous malignancy following prior cancer treatment is even rarer.^[9,10]^ Radiation-induced osteosarcomas, a recognized late complication of radiotherapy, typically manifest after a latency of 10 to 20 years, often in previously irradiated fields. Verrucous carcinoma, a well-differentiated squamous cell carcinoma variant, is another uncommon head and neck malignancy known for local invasiveness but low metastatic potential. The coexistence of these 2 primaries in the same anatomical region challenges current understanding of carcinogenesis and treatment paradigms, particularly in geriatric populations. While surgical resection remains the primary treatment for head and neck osteosarcoma, the role of adjuvant systemic therapies continues to evolve, with ongoing debates regarding their efficacy in preventing recurrence, especially in rare geriatric cases. Recent literature on sarcomas, including uterine leiomyosarcoma as a model, highlights the limited benefits of adjuvant chemotherapy in early-stage disease, as evidenced by meta-analyses showing no significant reduction in recurrence rates compared to observation alone.^[11]^ Genomic profiling of sarcomas has revealed key mutations in tumor suppressor genes like TP53 and RB1, which may drive tumorigenesis and serve as potential predictors of therapeutic response.^[12]^ Furthermore, the integration of novel immuno-oncology approaches, such as immune checkpoint inhibitors, is emerging as a promising avenue for advanced or metastatic sarcomas, although their application in head and neck osteosarcoma requires further investigation due to the tumor’s immunosuppressive microenvironment.^[13]^ Predictors of response, including inflammatory markers like the neutrophil-to-eosinophil ratio and cachexia index, have shown prognostic value across various cancers, including sarcomas, with elevated neutrophil-to-eosinophil ratio and low cachexia index associated with poorer survival outcomes, potentially guiding personalized treatment strategies.^[14,15]^ These advancements underscore the need for tailored adjuvant regimens that balance efficacy with toxicity, particularly in elderly patients with comorbidities.

This report presents a unique case of metachronous head and neck osteosarcoma in a 91-year-old patient with a history of verrucous carcinoma, aiming to elucidate diagnostic complexities, explore potential etiological links, and contribute to the sparse literature on geriatric oncology.

## 2. Case presentation

**Figure 1.**
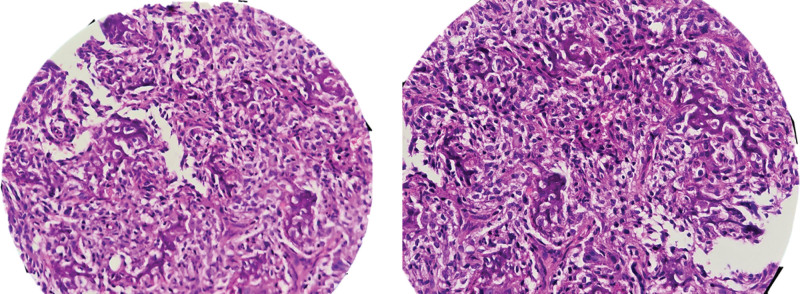
Axial CT of the neck at C1 (A) and mid-neck (B), showing bony and soft tissue structures.

**Figure 2.**
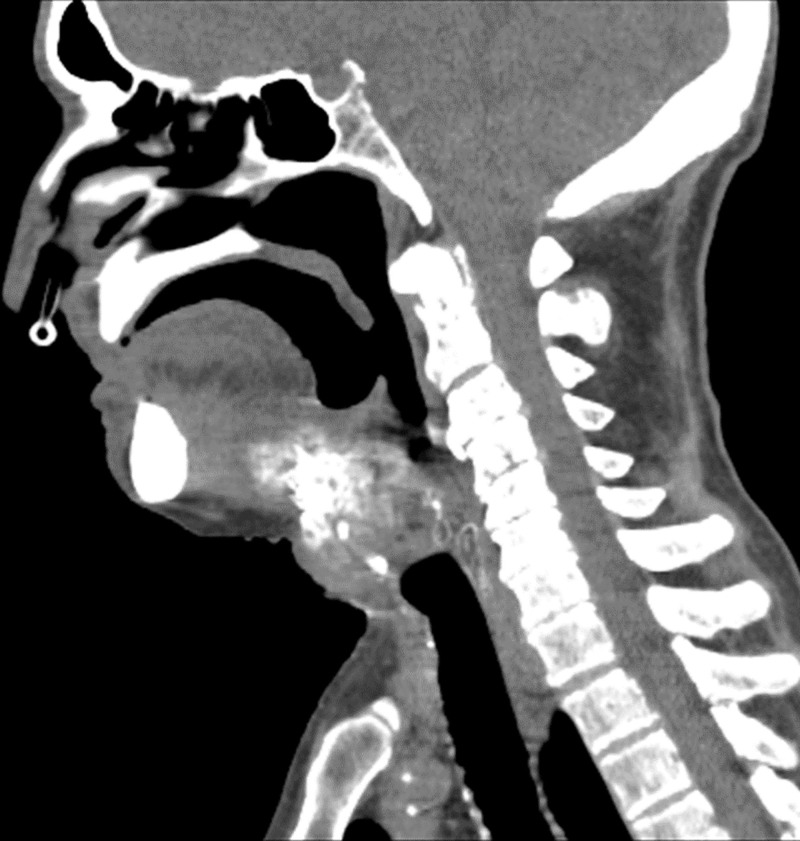
Sagittal CT demonstrating a calcified subcutaneous mass at the hyoid level, extending to the thyroid cartilage and tongue base.

**Figure 3.**
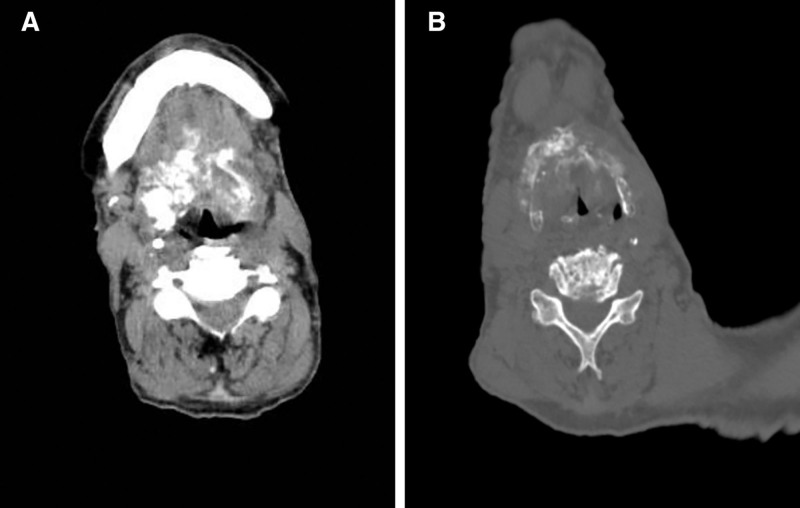
Histopathological examination reveals a heterogeneous neoplastic cell population comprising intermediate to large-sized cells exhibiting marked cellular atypia and pleomorphism. The neoplastic cells demonstrate characteristic malignant features, including nuclear hyperchromasia, irregular nuclear contours, and prominent nucleoli. These cells are encased within an extensive network of coarse, anastomosing trabeculae of neoplastic bone formation, displaying the classical “lace-like” pattern characteristic of osteosarcoma.

In 2006, a 91-year-old male presented with a 3.2 × 2.0 × 1.8 cm fungating mass on the right lateral tongue. Surgical excision confirmed verrucous carcinoma via histopathological examination, revealing hyperkeratosis, acanthosis, papillomatosis, and rete peg extension into the submucosa, with an involved lateral margin. Given the patient’s age, tumor location, and margin status, the multidisciplinary tumor board recommended definitive radiotherapy. He received 50 Gy in 25 fractions via intensity-modulated radiation therapy to spare adjacent structures, completing treatment in 2006. Follow-up occurred annually until 2011, with no recurrence noted; subsequent oncologic visits ceased until 2024. In 2024, the patient reported progressive hoarseness and dysphagia over 6 months. His medical history included hypertension and type 2 diabetes mellitus, managed with lisinopril and metformin, respectively, and no family history of cancer. Physical examination revealed a firm, non-tender neck mass. Laryngeal biopsy yielded a 1.5 × 1.0 × 0.5 cm specimen showing keratosis without dysplasia, characterized by squamous epithelium with acanthosis, parakeratosis, and mild stromal lymphocytic infiltration. Concurrently, a core needle biopsy of the neck mass produced fragments up to 1.0 × 0.1 cm, revealing a mesenchymal neoplasm with large, pleomorphic nuclei, high mitotic activity, osteoid production, and focal necrosis. Immunohistochemistry showed negative staining for cytokeratin and p63, with Ki-67 positivity in 20% of tumor cells, confirming osteosarcoma. Imaging corroborated these findings (Figs. 1 and 2), and histopathology demonstrated the classic “lace-like” osteoid pattern (Fig. 3). Radiotherapy was reinitiated in 2024 (30 Gy in 15 fractions), avoiding chemotherapy due to the patient’s frailty.

## 3. Discussion

This case illustrates a rare metachronous head and neck osteosarcoma in a 91-year-old patient, 18 years after radiotherapy for tongue verrucous carcinoma, highlighting diagnostic and therapeutic challenges in geriatric oncology. Head and neck osteosarcoma constitutes 6% to 10% of all osteosarcomas, predominantly affecting the mandible and maxilla in younger cohorts.^[1]^ Its presentation in elderly patients, especially as a secondary malignancy, is exceptional, with fewer than 20 reported cases in patients over 80.^[16]^ The 18-year latency aligns with radiation-induced osteosarcoma timelines (median 12–20 years^[6]^), suggesting a causal link to the 2006 radiotherapy field encompassing the neck. Verrucous carcinoma, diagnosed in 2006, is a low-grade malignancy with a favorable prognosis posttreatment, yet its management with radiotherapy may have predisposed this patient to a secondary neoplasm. Radiation-induced osteosarcomas arise in irradiated bone or soft tissue, often exhibiting aggressive behavior and poor outcomes compared to primary osteosarcomas.^[17]^ Here, the neck mass’s involvement of the hyoid, thyroid cartilage, and tongue base (Fig. 2) precluded surgical resection, favoring palliative radiotherapy. This decision reflects the balance between tumor control and quality of life in elderly patients, where comorbidities (hypertension, diabetes) and frailty limit aggressive interventions like chemotherapy (e.g., doxorubicin, cisplatin), standard in younger cohorts. Diagnostically, distinguishing osteosarcoma from other mesenchymal tumors (e.g., chondrosarcoma) required immunohistochemistry, with negative cytokeratin/p63 and positive Ki-67 (20%) confirming the diagnosis. The concurrent laryngeal keratosis, though benign, complicated initial assessment, emphasizing the need for multiple biopsies in complex cases. This metachronous presentation raises questions about carcinogenesis mechanisms—radiation effects, field cancerization, or age-related genomic instability. While genetic predisposition was absent, prior radiation likely played a pivotal role, supported by the tumor’s location within the original field. This case adds to the literature by documenting a rare geriatric osteosarcoma, contrasting with its typical demographic and anatomical profile. It underscores the importance of long-term follow-up post-radiotherapy and the utility of advanced diagnostics in atypical presentations. Future studies should explore molecular markers (e.g., TP53, RB1) to predict secondary malignancies and develop age-specific treatment protocols, addressing the current reliance on extrapolated guidelines. From our perspective as clinicians and researchers in oncology, this study holds significant potential to raise awareness about the underrecognized risks of secondary malignancies in elderly survivors of head and neck cancers, particularly those treated with radiotherapy decades prior. It can inform clinical practice by encouraging extended surveillance protocols and multidisciplinary approaches tailored to geriatric patients, potentially improving early detection and outcomes in similar rare cases. However, substantial knowledge gaps persist, including the exact molecular pathways (e.g., DNA damage response alterations) that drive radiation-induced osteosarcomas in aging tissues, the lack of standardized guidelines for long-term monitoring in this demographic, and the scarcity of data on effective, low-toxicity therapies for frail individuals. Researchers can address these gaps through collaborative efforts such as multi-institutional registries for rare geriatric sarcomas, advanced genomic and proteomic analyses of archived tissues to identify predictive biomarkers, and randomized controlled trials focused on adapted regimens like hypofractionated radiotherapy or novel targeted agents. Looking ahead, we envision this area unfolding rapidly over the next 5 years, propelled by advancements in precision oncology—such as widespread adoption of liquid biopsies for early secondary tumor detection, AI-driven risk stratification models, and immunotherapies that could offer less invasive alternatives to traditional chemotherapy. These developments may shift the paradigm from reactive palliative care to proactive prevention, ultimately enhancing quality of life for elderly patients at risk.

## 4. Limitations

Despite its contributions, this work has several limitations inherent to case reports. Firstly, as a single-patient study, it lacks generalizability to broader populations and cannot establish causality between the prior radiotherapy and osteosarcoma development without comparative controls. Secondly, genetic testing (e.g., for radiation-signature mutations) was not performed due to resource constraints, limiting insights into etiological mechanisms. Thirdly, long-term follow-up data beyond the 2024 re-irradiation are unavailable, precluding assessment of treatment efficacy or survival outcomes. Finally, the retrospective nature introduces potential recall bias in historical details, and the absence of advanced imaging modalities (e.g., PET-CT) at initial presentation may have influenced diagnostic timelines. These constraints highlight the need for larger-scale studies to validate and expand upon our findings.

## 5. Conclusion

This case report presents a rare and complex presentation of metachronous osteosarcoma in the head and neck region of a 91-year-old patient with a previous history of verrucous carcinoma. The case highlights several critical aspects of geriatric oncology and rare tumor presentations. First, it demonstrates the importance of maintaining a high index of suspicion for second primary malignancies in elderly patients with previous cancer history, even when the new tumor type is uncommon for the age group. Second, it emphasizes the crucial role of comprehensive histopathological evaluation and immunohistochemical studies in achieving accurate diagnosis, particularly in cases with unusual presentation patterns. Third, it underscores the unique challenges in managing elderly patients with complex oncological histories, where standard treatment protocols may need significant modification. The case also reveals significant gaps in our current understanding of head and neck osteosarcomas in elderly patients, particularly regarding optimal treatment strategies and prognostic factors. Future research should focus on developing age-specific treatment guidelines and identifying molecular markers that could guide therapeutic decisions in this vulnerable patient population. This case contributes to the limited literature on metachronous malignancies in elderly patients and serves as a valuable reference for clinicians managing similar complex cases in geriatric oncology. This case also highlights the potential long-term consequences of radiation therapy, emphasizing the need for extended follow-up in patients who have received radiation treatment to the head and neck region.

## Author contributions

**Investigation:** Abolfazl Koozari, Mohammad Reza Khademi.

**Methodology:** Mohammad Reza Khademi.

**Supervision:** Mohammad Reza Khademi.

**Writing – original draft:** Abolfazl Koozari, Mohammadreza Elhaie, Mohammad Reza Khademi.

**Writing – review & editing:** Abolfazl Koozari, Mohammadreza Elhaie, Mohammad Reza Khademi.
